# Core-Level Photoelectron Angular Distributions from Bulk-Solvated to Surface-Active Aqueous Potassium Carboxylate Salts

**DOI:** 10.1021/acs.jpcb.5c03673

**Published:** 2025-09-10

**Authors:** Tamires M. Gallo, Kalil Cristhian Figueiredo Toledo, Georgia Michailoudi, Ricardo dos Reis Teixeira Marinho, Olle Björneholm, Noelle Walsh, Gunnar Öhrwall

**Affiliations:** † Division of Synchrotron Radiation Research, Department of Physics, Lund University, P.O. Box 118, SE-221 00 Lund, Sweden; ‡ Institute of Chemistry, University of São Paulo—IQ-USP, Av. Lineu Prestes 748, 05508-000 São Paulo, São Paulo, Brazil; § Nano and Molecular Systems Research Unit, 6370 University of Oulu, P.O. Box 3000, FI-90014 Oulu, Finland; ∥ Institute of Physics, Brasilia University (UnB), 70.910-900 Brasília, Brazil; ⊥ Institute of Physics, Federal University of Bahia, 40.170-115 Salvador, Bahia, Brazil; # Department of Physics and Astronomy, Uppsala University, P.O. Box 516, SE-75120 Uppsala, Sweden; ¶ MAX IV Laboratory, Lund University, P.O. Box 118, SE-22100 Lund, Sweden

## Abstract

Photoelectron angular distributions are reported for a series of aqueous potassium carboxylate solutions, ranging from bulk-solvated to strongly surface-active species. The quantitative information determined from this work demonstrates how the measured photoelectron angular distributions are influenced by the ions’ increasing propensity for the surface in aqueous solutions. Our study provides insight into the relative depth and location of the carboxylate functional group, which is valuable for investigating the adsorption of organic molecules at liquid–vapor interfaces. This work provides a further demonstration that the liquid microjet technique employed together with the measurement of photoelectron angular distributions presents a promising avenue for determining the electronic and surface properties of solutes in liquid water and at the liquid–vapor interface, information that has potential application in fields such as atmospheric chemistry, biophysics, and materials science.

## Graphical abstract

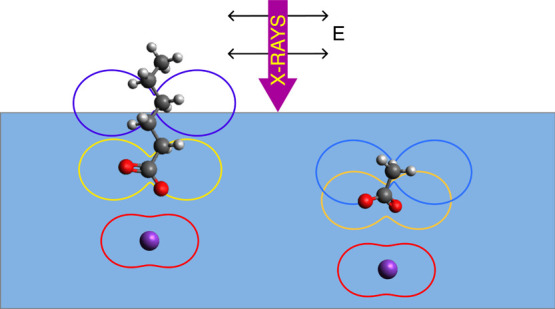

## Introduction

An in-depth understanding of electronic structure and bonding in aqueous solutions is critical for several areas of science and technology, including catalysis, environmental science, and biophysics.^1–5^ Carboxylate salt solutions are used in many such applications. For example, potassium formate is used as an activation agent when producing ultrathin graphene paper for green/sustainable energy devices with carbon-based supercapacitors.^6^ Carboxylate salts have also been used as model systems in many investigations of the surface properties of aqueous solutions with relevance to atmospheric chemistry.^7,8^

However, despite its relevance in various areas of applied research, the measurement of accurate electronic structure information for liquid samples is still quite demanding, because of the complexity of the condensed phase and the significant experimental challenges that arise when working with liquids in a vacuum. Fortunately, the development of the liquid microjet has proven particularly suitable for electron spectroscopy studies of volatile liquid samples, particularly for aqueous solutions.^9^ Liquid-jet photoelectron spectroscopy has enabled studies offering new insights into the electronic properties of solvated species and their role in chemical reactions in solution. Nevertheless, one detail that still deserves more attention is the effects of photoelectron angular distributions, which are interesting in their own right but can complicate the interpretation of experimental data.

Due to its inherent surface sensitivity, photoelectron spectroscopy is a powerful technique that can offer new insights into studies of liquid surfaces, including effects from photoelectron angular distributions.^10–13^ However, the photoelectron angular distributions from condensed phase samples are affected by elastic and inelastic scattering, which both are poorly constrained, making their use for depth profiling of liquid samples a complex matter.^10^ Still, surface-adsorbed species will in both aspects be affected less, because of the emitted electron’s shorter path in the condensed phase. Although several studies have investigated the angular distributions from valence and core orbitals in liquid water,^14–17^ fewer studies have focused on the angular distributions of electrons emitted from solutes, as such measurements are complicated by the surface propensity and distribution of the solute.^10–13^

Various experimental techniques, including surface tension measurements and photoelectron spectroscopy, have been used to investigate the surface activity of solutes in aqueous solutions. Previous photoelectron spectroscopy studies on carboxylate salts in aqueous solution have shown that shorter-chain carboxylates, such as formate, are bulk solvated, while longer-chain carboxylates lead to an increased tendency for surface enrichment.^8,18–21^ In accordance, surface tension measurements have shown that sodium formate increases the surface tension of aqueous solutions,^22,23^ while a surfactant such as propanoate or butanoate decreases it.^23^ In other words, increasing the alkyl chain length of the carboxylate leads to an increased tendency for concentration at the surface. Clearly, carboxylate salts are good model systems with which to investigate the influence of surface propensity on measured photoelectron angular distributions in aqueous solutions.

From experimentally measured photoelectron angular distributions (PADs), Dupuy et al. investigated the adsorption of octanoic acid at the liquid–vapor interface of aqueous solutions.^10^ The orientation of octanoate molecules was found to be more perpendicular to the surface compared to that of the octanoic acid molecules at the liquid–vapor interface of a solution. Furthermore, the carboxylate groups were determined to be located deeper into the bulk than the carboxylic acid groups, thus providing valuable insight into the relative depth/location of the carboxylate and carboxylic acid functional groups in solutions with varying pH. Basically, adsorption at the surface can be understood to lead to an increase in the anisotropy of PADs due to the decreased elastic scattering, which may provide a new approach to investigate the adsorption behavior of solutes at liquid–vapor interfaces.^10,11^ More research is needed to understand the details of this effect and to determine its general applicability to other solutes.

For spherically symmetric or randomly oriented systems (i.e., free atoms or free molecules, respectively), the beta formalism can describe the angular distribution of photoelectrons.^24^ Random orientation can even be assumed with some confidence for bulk-solvated species in solution. However, surface-adsorbed species cannot generally be considered to be randomly oriented, and therefore, this description may not always be suitable. In the dipole approximation, the following equation describes the photoelectron differential cross section for a randomly oriented system excited by plane-polarized radiation^25^

1 $$\frac{d\sigma }{d\Omega }=\frac{\sigma }{4\pi }\left(1+\frac{\beta }{2}\left(3\cos^{2}\theta -1\right)\right)$$

where σ is the integrated photoionization cross section, Ω is the solid angle of detection, β is the anisotropy parameter and θ is the angle between the direction of polarization and the emitted electron.

To learn more about the surface propensity of carboxylate ions, we have studied photoelectron angular distributions for a series of aqueous potassium carboxylate solutions, ranging from bulk-solvated (formate) to strongly surface-active species (hexanoate). We have used the description presented in eq 1, assuming an essentially random distribution even for surface active species, as Dupuy et al. did for octanoate.^10^ Our results clearly show that the photoelectron angular distributions of surface-adsorbed species are less affected by elastic and inelastic scattering due to their shorter path in the condensed liquid. This work emphasizes the importance of considering the influence of the surface propensity of solutes on photoelectron spectroscopy data from aqueous solutions.

## Experimental Methods

The experiments were performed at the FlexPES beamline on the 1.5 GeV ring, MAX IV Laboratory in Lund, Sweden. FlexPES provides suitable facilities for high-resolution photoelectron spectroscopy, using linearly polarized radiation from a planar undulator in the photon energy range 40–1500 eV.^26^ During these experiments, the storage ring was operated in “top-up” mode, generating a nearly constant photon flux at a ring current of approximately 400 mA. The photoelectron spectroscopy experiments were performed using a Scienta R4000 electron spectrometer installed in the Low-Density Matter (LDM) photoemission endstation. The endstation is designed such that the spectrometer can rotate around the photon beam in the plane perpendicular to it, thus allowing for angle-resolved photoelectron spectroscopy measurements. The measurements were performed during two different campaigns: formate and acetate were studied in one campaign, and propanoate, butanoate, and hexanoate in another.

The liquid microbeam was generated using a liquid microjet setup with a commercially purchased 24 μm diameter nozzle (Advanced Microfluidic Systems GmbH). The liquid microjet was injected into the vacuum chamber perpendicular to the photon beam and the spectrometer lens axis using a flow rate of 0.6 mL/min, achieved using HPLC pumps. The first experiments (on formate and acetate) employed a LabAlliance, Series III pump, and the later experiments (on propanoate, butanoate, and hexanoate) employed a Knauer Blueshadow 40P pump. A BIOTECH DEGASi PLUS Semi-Prep degasser system was installed in the liquid lines to reduce the amount of dissolved gas in the fluid stream. The liquid jet was mounted in a differentially pumped compartment, an essential feature of the setup to ensure that a vacuum of approximately 2 × 10^–5^ mbar or lower is maintained in the spectrometer chamber. The liquid jet intersected the radiation from the beamline at the laminar part of the flow, around 2–3 mm after exiting the nozzle, before the jet breaks up into droplets. The liquid sample was then collected in a liquid-nitrogen-cooled trap where it was frozen so that a reasonable pressure could be achieved in the interaction chamber. Electrons ejected from the sample as a result of interaction with the synchrotron light passed through a skimmer cone into the spectrometer. The skimmer was positioned close to the liquid surface with the nominal distance between the jet and the cone ∼2 mm for the first set of experiments and ∼1 mm for the second set of experiments. Two different skimmer cones were used for these experiments, each of which had an opening diameter of 0.5 mm and an acceptance angle of approximately 14° and 28°, respectively.

During these measurements, the beamline exit slit was open to 100 μm, which results in photon bandwidths of 130 meV–240 meV for the photon energy range used in these experiments. The Scienta R4000 analyzer was operated with a pass energy of 200 eV, and a 0.5 mm wide slit was used, resulting in a spectrometer resolution of 250 meV. The spectra were recorded with photon energies from 360 to 550 eV (360, 380, 400, 425, 450, 500, 550 eV), for the K 2p and C 1s core lines, with binding energies of approximately 300 and 290 eV, respectively. The O 1s of the carboxylate group was not studied, due to the large overlap with the much stronger signal from the solvent.

To compare bulk solvated K^+^ ions and carboxylate anions with varying surface propensity, the measurements were done at two angles, the “magic angle” (≈54.7°) and 90°, relative to the horizontal polarization of the synchrotron radiation, over a range of photon energies. As eq 1 shows, at the “magic angle” the measured intensity is proportional only to the integrated photoionization cross section, and any anisotropy in the angular distribution does not come into play. Initially, the experimental measurements were conducted at an angle of 54.7° for all energies. Subsequently, the angle was changed to 90°, and the second set of data was acquired using the same experimental parameters. The detection angle was set using a graded scale on the rotary stage and checked using an inclinometer, resulting in an estimated uncertainty of ±1° for the central angle relative to the horizontally polarized radiation. The spectrometer has an angular acceptance, which is considerably larger than the possible error in the central detection angle, and using eq 1 for the central angle will lead to a slight underestimate of the anisotropy. The angular acceptance of the spectrometer is different in the dispersive and nondispersive directions, with a nontrivial dependence on kinetic energy, and we have for simplicity neglected its influence.

For the first experimental session, aqueous solutions of 1.0 M (mol/L) potassium acetate (KCH_3_COO; BioXtra, Sigma-Aldrich, ≥99.0% purity) and potassium formate (KHCOO; Reagentplus (R), Aldrich, 99% purity) were prepared using the chemicals without further purification and deionized water (Milli-Q, 18.2 MΩ cm). Estimates of pH values for the solutions were obtained using pH sticks (MColorpHast, pH 0–14, Merck) and found to be approximately 7 and 6, respectively. In this pH range, almost complete deprotonation of the carboxyl groups is expected. For the second experimental session, aqueous solutions of 0.5 M potassium propanoate (KC_2_H_5_COO), 0.25 M potassium butanoate (KC_3_H_7_COO), and 0.05 M potassium hexanoate (KC_5_H_11_COO) were prepared using a different method. The solutions were prepared by titrating the corresponding carboxylic acids (propanoic acid (Sigma-Aldrich, ≥99.5%), butanoic acid (Aldrich, ≥99%), and hexanoic acid purum (Sigma-Aldrich, ≥98.0%) with potassium hydroxide (0.1 M KOH; EMSURE, ≥85% purity). The resulting solutions had pH values of 8.7, 9.42, and 8.67, respectively, as measured using a pH meter (Mettler Toledo—FiveEasy plus FP20). Using published values for the p*K*_*a*_ of the corresponding carboxylic acids,^27^ the estimated pH values of the potassium carboxylate solutions would be 9.28 for 0.5 M propanoate, 9.11 for 0.25 M butanoate, and 8.77 for 0.05 M hexanoate. The small deviations in pH of the experimentally prepared solutions mean that the imbalance in potassium to carboxylate ions is negligible, at most ∼10^–5^ M. All solutions were prepared using standard deionized water (Milli-Q) and filtered before measurement using 1.2 μm Whatman Puradisc FP30 syringe filters to remove any solid particles.

The spectra were fitted to obtain the areas of the core-level peaks and of the background on top of which the peaks sit. Details of the curve fitting procedure can be found in the Supporting Information. In the Supporting Information, the peak areas and the derived β values (see below for a discussion of the normalization procedure) for the formate, acetate, propanoate, butanoate, and hexanoate solutions are presented in Tables S1–S5, respectively.

## Results and Discussion

Figure 1 displays photoelectron spectra of the K 2p and C 1s core levels of aqueous potassium acetate (KCH_3_COO) obtained at two different angles relative to the horizontally polarized radiation (θ = 54.7° dashed lines and 90° solid lines). The spectra were collected at photon energies between 360 and 550 eV, and the binding energy has been calibrated to published values for C 1s in aqueous sodium acetate.^18^ The peaks located at ∼301 eV and ∼298 eV are attributed to K 2p_1/2_ and K 2p_3/2_, respectively, while the peaks at ∼293.4 eV and ∼289.9 eV correspond to C 1s of COO and CH_3_, respectively.

**Figure 1:**
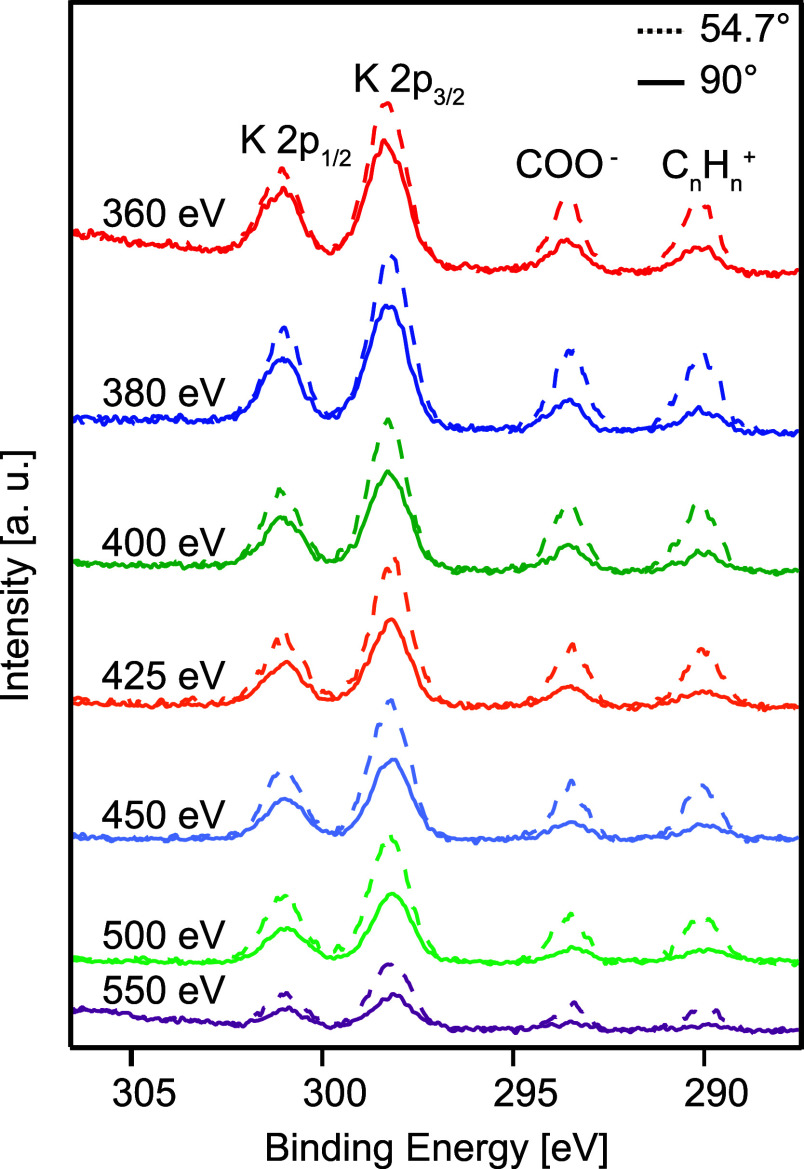
C 1s spectra from potassium acetate (KCH_3_COO) recorded at 54.7° (dashed line) and 90° (solid line), for photon energies of 360, 380, 400, 425, 450, 500, and 550 eV.

Similar spectra were recorded for potassium formate, propanoate, butanoate, and hexanoate. In all spectra, the C 1s binding energies of the aliphatic C_*n*_H_*m*_ carbons nearly coincide, forming a single peak, well separated from the C 1s of COO^–^. All spectra were normalized for acquisition time (i.e., number of sweeps), and at each energy, the spectrum obtained at 54.7° was scaled by a factor based on the ratio of the areas below the linear backgrounds used in the fitting procedure at 54.7° and 90°. This normalization is rationalized by the assumption that the background, originating from scattered electrons from valence band ionization and Auger transitions, comes from the same source volume as the core-level photoelectron lines. Although inelastic scattering will lead to different mean escape depths of the background and core-level electrons, they will be affected by experimental effects, such as the photon flux, the alignment of the photon beam and the liquid jet, and the transmission of the spectrometer in the same way.

As seen in Figure 1, the intensity of the C 1s peaks relative to the K 2p peaks is lower in the spectra recorded at 90°, showing a greater angular anisotropy for the C 1s electrons than those from K 2p ionization, as expected from calculated atomic differential cross sections.^28^ With the normalization used, the β value can be deduced from the peak area ratios if an assumption about the angular distribution for the background is made, e.g., that the background is isotropic. In a recent study of aqueous ammonium nitrate,^13^ we used simulations to show that the background after N 1s ionization with similar kinetic energies exhibited a low positive β value (around 0.3). In the present case, the kinetic energy difference between the valence band electrons (the main source of scattered electrons, mostly from the solvent) and the core levels in question is smaller, and fewer scattering events have likely occurred to reduce the kinetic energy to be close to that of the core levels, which could point to a higher β value for the background than for the case of N 1s. In the spectrum recorded at 550 eV, the C 1s and K 2p photoelectron peaks overlap with Auger features from both carbon and potassium, for instance the K L_2,3_M_2,3_M_2,3_ is seen near the binding energy ∼305 eV (kinetic energy ∼245 eV). The fact that the Auger peak intensities at 90° and at 54.7° nearly coincide when normalizing to the background signal is strong evidence that the background is close to isotropic at this photon energy, since the angular anisotropy of both atomic and molecular Auger emission is expected to be low far from the threshold,^29–31^ and for the condensed phase it will be even more isotropic due to elastic scattering. We have selected to present the β values obtained by assuming an isotropic background, as the simplest approach, while acknowledging that it may be a slight underestimate based on experience from the ammonium nitrate case.^13^

Before turning to the angular anisotropy parameter β, we will discuss the ratios of the peak areas for the spectra recorded at the “magic angle”, 54.7°. These ratios do not rely on any assumption on the properties of the background and are therefore a more robust quantity than the β value. Assuming the same C 1s photoionization cross section for all carbon atoms, a stoichiometric ratio of the C 1s peak intensities would be expected in the absence of inelastic scattering at the magic angle (54.7°). The graph in Figure 2 shows the ratio of the C 1s peak areas of the aliphatic carbons (C_*n*_H_*m*_) and the carboxylate carbon (COO^–^) at 54.7° for different potassium salts plotted against the photon energy (*h*ν) (acetate [KCH_3_COO—blue mark], propanoate [KC_2_H_5_COO—green mark], butanoate [KC_3_H_7_COO—purple mark], and hexanoate [KC_5_H_11_COO—black mark]).

**Figure 2:**
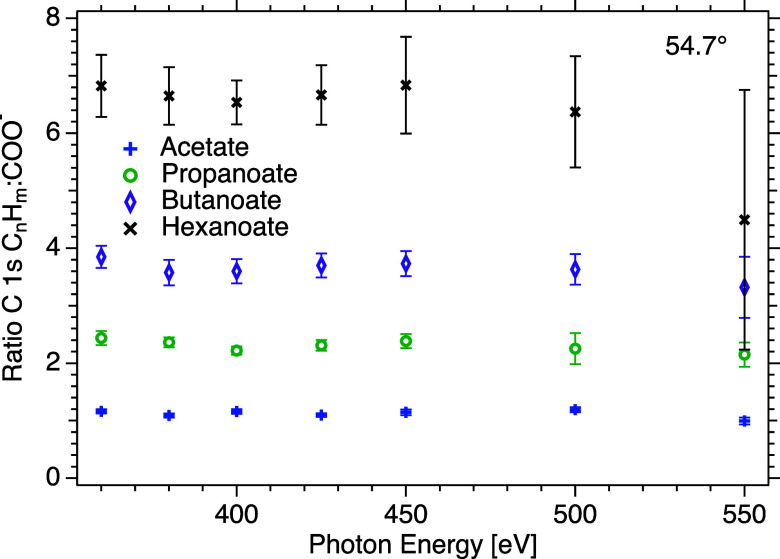
C 1s peak-area ratio of C_*n*_H_*m*_: COO^–^, obtained from spectra recorded at 54.7°, as a function of photon energy, for potassium hexanoate (KC_5_H_11_COO—black mark), butanoate (KC_3_H_7_COO—purple mark), propanoate (KC_2_H_5_COO—green mark), and acetate (KCH_3_COO—blue mark). Note that the error bars represent statistical uncertainties only.

The ratios for KCH_3_COO, KC_2_H_5_COO, and KC_3_H_7_COO are relatively constant around ∼1.1, ∼2.4, and ∼3.8, respectively, with only a weak dependence on photon energy. For KC_5_H_11_COO, the ratio is ∼6.8 at the lowest photon energy, but at the highest energy, *h*ν = 550 eV, it is considerably lower, even below the stoichiometric ratio (≈4.4). Note that for this particular energy and sample, the error bar is very large because the COO^–^ peak in the spectrum was very weak and that the peaks sit on top of the background for the O 1s Auger decay from the solvent, giving unfavorable signal-to-noise ratios. The peak area ratios are larger than that expected from stoichiometry, which can be rationalized by the orientation of the molecules at the surface, with the aliphatic chains tending to point outward and the carboxylate group further in, so that inelastic scattering suppresses the carboxylate carbon signal more than that from the carbon atoms in the chains. The ratios at *h*ν = 400 eV can be compared to those obtained with another setup: 1.19, 3.63, and 6.80 for sodium acetate, butanoate, and hexanoate,^8^ compared to 1.16, 3.60, and 6.54 in our measurement, which agree well within our error bars. Note that the salt concentration in the other study was 0.1 M for all solutions, less than ours for acetate and butanoate but higher than ours for hexanoate, which may affect the values to some degree.

Assuming an inelastic mean free path (IMFP) of λ ≈ 1 nm, similar to that of liquid water in the kinetic energy range studied here,^15,32^ an increased path of ∼λ·ln­(6.8/5) ≈ 0.31 nm can be estimated for electrons from carboxylate carbon compared to the chain in the case of hexanoate (see Supporting Information). This is comparable to the distance between the carboxylate carbon and the central carbon in the aliphatic chain, ≈0.39 nm,^33^ which in the simplest interpretation would point to an orientation of the molecule with the carboxylate group farther away from the surface than the chain and a tilt angle of θ ∼ 50°. However, for a quantitative understanding, a more elaborate model must be used, e.g., considering distributions of orientations and conformers, relying on Monte Carlo simulations for the transport properties, and taking into account the uncertainty in the value of the IMFP. However, we are convinced that this simple picture can still give a qualitative gauge of the orientation of the molecules in the surface region. Using the same approach for the other cases, increased paths of ≈0.10 nm, ≈0.18 nm, and ≈0.24 nm are estimated for the carboxylate carbon atom in acetate, propanoate, and butanoate, respectively, which are comparable to the distances between the carboxylate carbon atom and the central point in the aliphatic chain.

Figure 3 shows the peak area ratio of COO C 1s/K 2p_3/2_ at an angle of 54.7°, following the same color pattern as in the previous graph, but including potassium formate (KHCOO—red symbols). The ratio of calculated atomic photoionization cross sections of C 1s and K 2p_3/2_ ^28^ is also plotted as a dashed line, to indicate the expected photon energy dependence. The experimental ratios show a dependence on photon energy, which is more pronounced than in the case of the C 1s peak areas of (C_*n*_H_*m*_)­(COO^–^) presented above, and does not correspond to the behavior of the ratio of the calculated atomic cross sections. The IMFP is expected to increase with kinetic energy, meaning that the ratio should tend toward the ratio of the cross sections with increasing photon energy, and a weakly decreasing trend can be observed above 400 eV. The kinetic energies of the electrons from potassium and carbon are similar, and therefore, the transport properties due to inelastic and elastic scattering would be expected to be comparable, so the varying behaviors for the ratio up to ∼400 eV could possibly be due to intramolecular scattering phenomena, such as has been observed in free molecules.^34,35^

**Figure 3:**
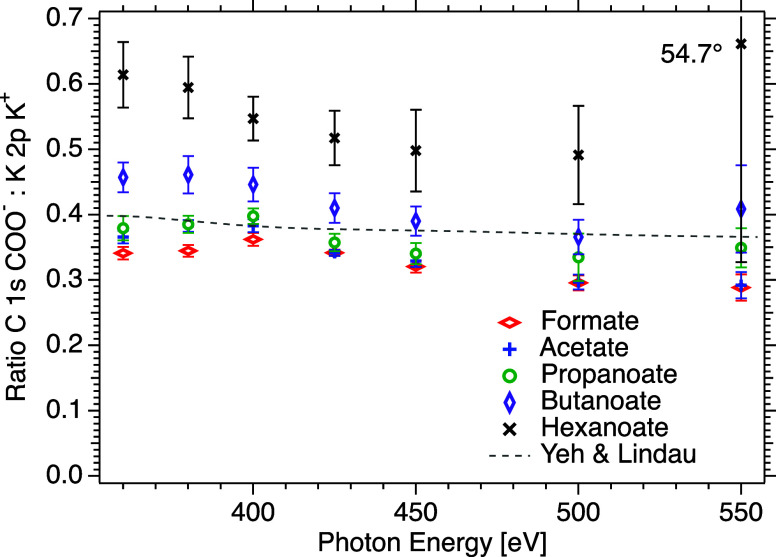
Peak-area ratio of COO^–^ C 1s/K^+^ K 2p_3/2_ obtained from spectra recorded at 54.7° as a function of photon energy, for solutions of potassium hexanoate (KC_5_H_11_COO—black mark), butanoate (KC_3_H_7_COO—purple mark), propanoate (KC_2_H_5_COO—green mark), acetate (KCH_3_COO—blue mark), and formate (KHCOO—red mark). Note that the error bars represent statistical uncertainties only. Calculated values for atomic carbon and potassium are included for reference (dashed line).^28^

In all cases, the stoichiometric ratio of potassium and carboxylate carbon is the same (1:1), but a clear increase in the ratio of the peak areas can be seen with increasing chain length. Potassium ions are expected to avoid the surface, so this observation clearly shows that the carboxylate group comes closer to the surface than the potassium ion for the surfactants with the longest chains.

We can, in the same way as discussed above for the ratio of the carboxylate carbon and the aliphatic chain carbons, derive a number gauging the separation of the carboxylate carbons and the potassium ions in the solution. We assume an exponential decay and the same IMFP for the K 2p and C 1s electrons, which is not unreasonable since the kinetic energy difference is small, and the transport properties of the medium the electrons are traveling through will not differ dramatically as a function of depth. For the most extreme case, i.e., for hexanoate, the ratio is approximately 1.5–1.8 as high as for formate, where we can assume that the potassium and carboxylate ions are nearly evenly distributed. Assuming an IMFP of λ ∼ 1 nm, an increased path of ∼λ·ln(1.5–1.8) ≈ 0.4–0.6 nm can be estimated for the electrons from the potassium ions compared to the electrons from the carboxylate carbon in the potassium hexanoate solution.

In a recent paper, where careful measurements of the absolute intensity of the C 1s peaks of carboxylate and alkyl-ammonium salt solutions were presented, the data shows how the intensity of the C 1s peak of the carboxylate carbon increases with chain length, which is understood as an effect of the ion moving closer to the surface.^8^ In that paper, sodium was used as the counterion of the carboxylate anion, but we would not expect any major difference for the case discussed here, where potassium ions were used. The increase in intensity of the C 1s peak of the carboxylate group between formate and hexanoate is approximately 7-fold at *h*ν = 400 eV,^8^ much greater than the relative increase in the signal of the counterion that we have observed for the same carboxylate ions. We therefore conclude that the potassium ions must have been dragged closer to the surface by carboxylate ions, from electrostatic interactions. At *h*ν = 400 eV, the COO C 1s/K 2p_3/2_ peak area ratio was 0.36 for formate and 0.55 for hexanoate, and assuming the same increase in the carboxylate signal as observed in the other study, this would correspond to an increase of a factor of ∼4.6 in the intensity of the potassium signal.

As noted above when discussing Figure 1, the assumption that the background has an isotropic distribution is supported by the observation that at *h*ν = 550 eV, Auger features, which are expected to have a nearly isotropic angular distribution, almost perfectly overlap when normalizing the data in this way. However, at this particular photon energy, the majority of the background intensity comes from scattered electrons from Auger decays of O 1s ionization of the solvent, which initially have an isotropic angular distribution. It may thus be that a low positive beta value should be expected for the background at lower photon energies, and if this is the case, the β values for the core levels would be slightly higher. In the case of N 1s ionization of NH_4_NO_3_, the increase was around ∼0.15 in the kinetic energy range 60 eV–120 eV,^13^ similar to the first few points in the current study.

In Figure 4, the beta values derived in this way for the carbons in the aliphatic chain of the carboxylate ions are presented. In all cases, an increase from values around 1.35–1.45 at the lowest photon energy to around 1.5–1.75 at the highest, seemingly tending toward an asymptote. There is considerable overlap of the error bars, but the data points for the longer chains are consistently higher than those for shorter chains, and clearly show that the beta value increases with chain length, which can be understood by reduced elastic scattering the further out the aliphatic chains protrude from the surface. We note that a value of β = 1.60 was obtained for C 1s of C_*n*_H_*m*_ at *h*ν = 450 eV in 0.1 M aqueous sodium octanoate,^10^ an even stronger surfactant than those studied here. This is slightly lower than the value we have obtained for hexanoate at the same photon energy (β = 1.67). A similar increasing behavior of the anisotropy parameter has been observed for C 1s in aqueous NaC_4_F_7_COO,^11^ N 1s in aqueous NH_4_NO_3_,^13^ and O 1s in liquid water.^15^ The beta values for the present C_*n*_H_*m*_ C 1s data are higher for *h*ν = 450 eV than those observed for the C 1s of the CF_*m*_ carbon atoms in perfluoropentanoate.^11^ The gas-phase value of β for the C 1s of CF_*m*_ atoms in perfluoropentanoic acid is considerably lower than that of C_*n*_H_*m*_ atoms in pentanoic acid, 1.66–1.67 ^11^ and 1.96,^10^ respectively, so the difference between the perfluorinated system and our data is not surprising.

**Figure 4:**
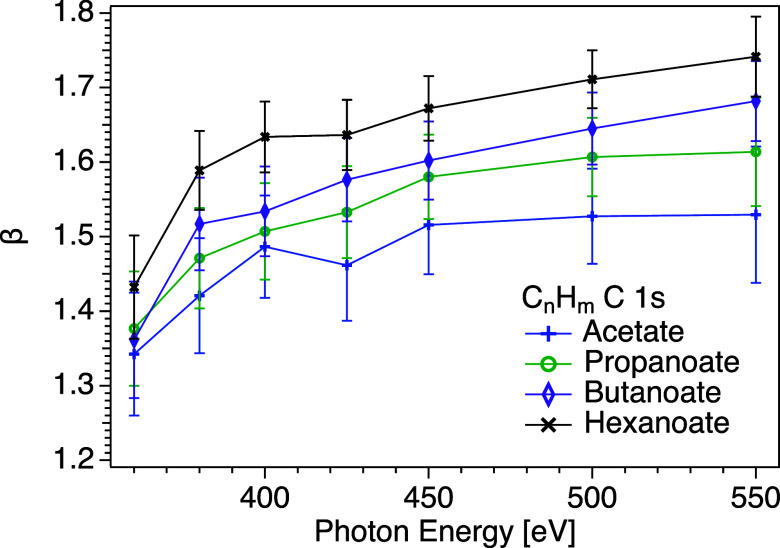
C 1s β values of C_*n*_H_*m*_ as a function of photon energy for solutions of potassium hexanoate (KC_5_H_11_COO—black mark), butanoate (KC_3_H_7_COO—purple mark), propanoate (KC_2_H_5_COO—green mark), and acetate (KCH_3_COO—blue mark). The lines are guides for the eyes. Tabulated data are available in the Supporting Information. Note that the error bars represent statistical uncertainties only.

We also note that the values increase relatively smoothly, with little evidence of deviation for the *h*ν = 550 eV point. This suggests that the assumption of a nearly isotropic background, which is supported by the C 1s Auger features observed for the *h*ν = 550 eV case, also has some validity for lower photon energies. As mentioned above, a positive beta value for the background would mean that the beta value for the core-level electrons would increase compared to the case with an isotropic background, and while the error bars are substantial, a dramatic deviation from β = 0 seems unlikely to us.

In Figure 5, C 1s β values of carbon in the COO^–^ group are presented for all carboxylate salts studied. A similar increasing trend to that seen in Figure 4 is observed, but the values are lower and the difference between the samples is less pronounced. There is a tendency for lower β values in the systems with no or short aliphatic chains compared to those with longer chains, which would be consistent with these being further from the surface and experiencing more extensive elastic scattering. However, considering the substantial overlap of the error bars, we cannot declare this definitively. The values for hexanoate are slightly higher than for the other samples, especially at lower photon energies, where the IMFP is the shortest and therefore the surface sensitivity is the largest. The lower value of the C 1s of the COO^–^ group compared to the C_*n*_H_*m*_ carbons is consistent with observations made in both the gas phase and the liquid phase.^10^ At *h*ν = 450 eV, a value of β = 1.41 was observed for COO^–^ in octanoate,^10^ and at the same energy, we observed values ranging from β = 1.38 for formate to β = 1.51 for hexanoate. As in the case of carbons in the chain, we observe a higher β value for the carboxylate group in hexanoate than Depuy et al. did for octanoate, which may indicate a systematic difference between the measurements.

**Figure 5:**
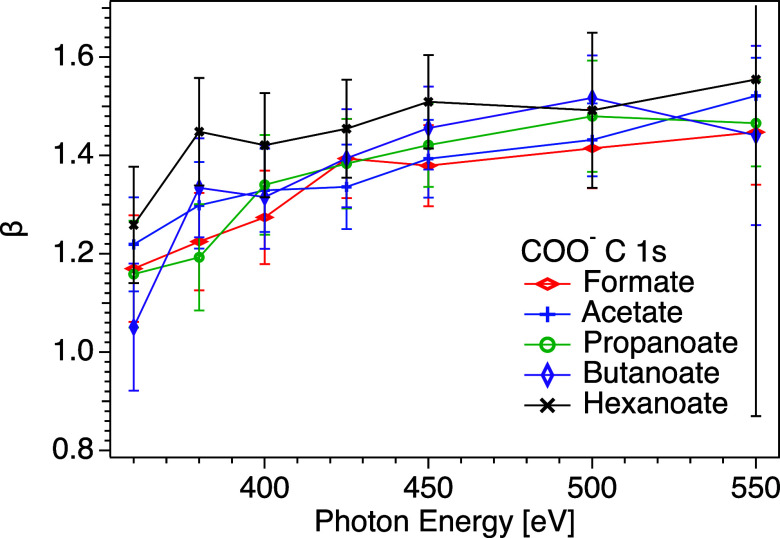
C 1s β values of the COO^–^ group as a function of photon energy, for solutions of potassium hexanoate (KC_5_H_11_COO—black mark), butanoate (KC_3_H_7_COO—purple mark), propanoate (KC_2_H_5_COO—green mark), acetate (KCH_3_COO—blue mark), and formate (KHCOO—red mark). The lines are guides for the eyes. Tabulated data are available in the Supporting Information. Note that the error bars represent statistical uncertainties only.

In Figure 6, the β values for K 2p_3/2_ for all potassium carboxylate salt solutions are presented as a function of the photon energy. We do not observe any obvious tendency for the beta values to depend on the chain length of the carboxylate counterion, except possibly in the case of hexanoate for the lower photon energies, where the deviation is still within the error bars. Together with the observation that the intensity of the potassium signal is expected to be higher for the stronger surfactants, which is strong evidence that the concentration in the surface region is higher for such samples as discussed above, the weak dependence on chain length shows that even for the surfactant samples, potassium gives an essentially bulk-like response in terms of the elastic scattering.

**Figure 6:**
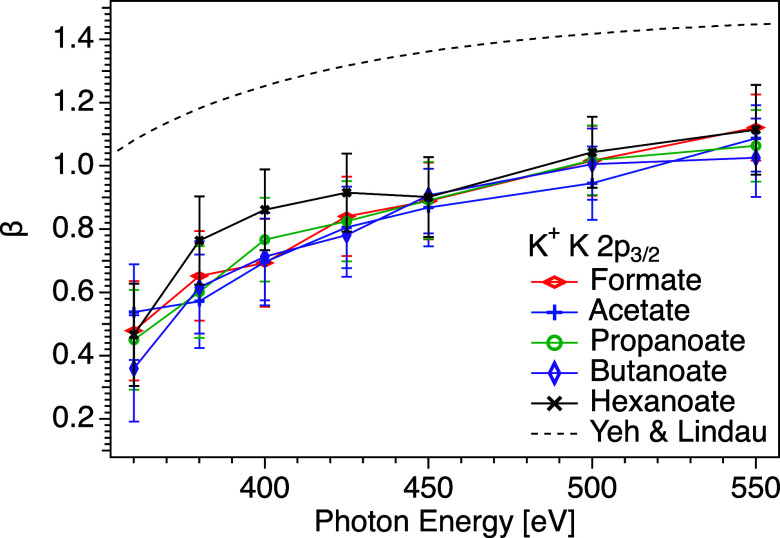
K 2p_3/2_ β values for potassium ions as a function of photon energy, for solutions of potassium hexanoate (KC_5_H_11_COO—black mark), butanoate (KC_3_H_7_COO—purple mark), propanoate (KC_2_H_5_COO—green mark), acetate (KCH_3_COO—blue mark), and formate (KHCOO—red mark). Calculated values for atomic potassium are included for reference (dashed line).^28^ The lines are guides for the eyes. Tabulated data are available in the Supporting Information. Note that the error bars represent statistical uncertainties only.

The ratio of the values of the liquid phase β to the value of the gas phase has been used to determine the depth distribution of ions in the surface region with Ångström resolution,^11^ and generally the ratio decreases with increasing elastic scattering when the atom is farther away from the interface. Unfortunately, we do not have data for gas-phase β values, but we have compared the results for the aqueous solutions to calculated atomic beta values.^28^ The ratio of the measured liquid phase β values and the calculated atomic β values increases from ∼0.4 to ∼0.7 between *h*ν = 360 eV and *h*ν = 550 eV for K 2p_3/2_. An increase in this ratio is not surprising, considering the expected decrease in the electron transport cross section in this kinetic energy range.^36^

The corresponding ratio for the β values of C 1s for the carbons in the chains lies in the range ∼0.68 at *h*ν = 360 eV to ∼0.76 at *h*ν = 550 eV for acetate and ∼0.72 to ∼0.87 for hexanoate, assuming a value of β = 2 for atomic C 1s electrons. For gas-phase pentanoic acid, a value of β = 1.96 was determined for chain carbons at *h*ν = 450 eV,^10^ so for at least the higher photon energies, this is not an unreasonable assumption. For carbons in the carboxyl group, intermediate values are obtained, ranging from 0.61 at *h*ν = 360 eV and 0.71 at *h*ν = 550 eV for acetate and 0.63 at *h*ν = 360 eV and 0.78 at *h*ν = 550 eV for hexanoate, again assuming a value of β = 2 for atomic C 1s electrons. A high, but slightly lower β value was observed for carboxyl carbon in gas-phase pentanoic acid, β = 1.87, than for chain carbons at *h*ν = 450 eV,^10^ which would mean a slightly higher ratio.

We clearly observe a similar trend, with a greater reduction in the β value for the atoms that are the furthest away from the surface. As the data presented here show, the determination of the angular distribution parameter β is a sensitive tool to investigate the surface distribution of molecules in aqueous solutions.

## Conclusions

We have investigated angular effects in photoemission from aqueous potassium carboxylate solutions, using a cylindrical liquid jet setup, by recording electron spectra at 54.7° and 90° relative to the horizontal polarization of the synchrotron radiation, with photon energies ranging from 360 to 550 eV.

From data recorded at 54.7°, where angular effects are minimized, we found that the ratios of the C 1s intensities of the C_*n*_H_*m*_ and COO^–^ peaks are generally higher than the stoichiometric ratio, indicating a preference for the C_*n*_H_*m*_ group to be oriented out of the surface. This tendency increases with the length of the chain. The ratio decreases with photon energy, approaching the stoichiometric value, as is expected from the increase in mean free path of the electrons with increased kinetic energy. The ratio of the C 1s signal of the carboxylate group to K 2p of the potassium ion at 54.7° shows a tendency to increase with chain length. This is consistent with a picture where the carboxylate anions are dragged closer to the surface by the aliphatic group, and the potassium counterions tend to reside further in.

Using the assumption that the inelastic background on which the C 1s and K 2p peaks reside has an isotropic distribution has allowed us to normalize the data recorded at 54.7° and 90°, and from this, we derive β values to describe the angular distributions. For data recorded at *h*ν = 550 eV, this assumption is supported by the fact that K 2p and C 1s Auger features, which are expected to have a nearly isotropic angular distribution, show a normalization ratio similar to the background. In addition, for the five samples for which we present data in this paper, we obtain consistent and physically reasonable results, which gives us confidence that this normalization procedure has some merit.

For the aliphatic carbons, we observed higher values for β than from the carboxylate carbon in all cases. By comparing the ratio of the obtained β values and the gas phase molecular data for a similar system,^10^ we conclude that the C 1s electrons from the aliphatic chains suffer less elastic scattering than those from the carboxylate group, consistent with the observation of the intensity ratio at 54.7°. The β value for the C 1s of the aliphatic carbons increases with chain length, showing an increased propensity of the surfactants to reside at the interface with the chain sticking out.

For the C 1s from the carboxylate group, there is only a weak tendency for higher β values for the longer chains, and almost completely within our error bars. Even for the strongest surfactants, the increased elastic scattering from residing slightly further in than the aliphatic chain carbons leads to a relatively marginal difference. For the K 2p electrons from the potassium ions, the difference is even smaller, with no clear tendency for a difference between the salts. This contrasts with the intensity ratio of the carboxylate carbon and potassium ion signals at 54.7°, where the C 1s/K 2p ratio was found to be significantly greater for the longest chains. To reconcile these observations, the sample density distribution in the surface region must be taken into account, for instance using results from MD simulations, which is beyond the scope of this study.

In conclusion, angular effects on photoemission are a sensitive probe of the distribution of ions in the vicinity of the surface. The combination of the liquid microjet technique and the measurements of PADs presents a promising technique for studying the electronic and surface properties of solutes in liquid water and at liquid–vapor interfaces.

## Supplementary Material



## Notes

Details about the fitting procedure used to analyze the data. Explanation of the estimate of the orientation of the molecules at the surface. Tables with experimental data derived from the fits (PDF)

## ∇

European Spallation Source ERIC, Box 176, SE-221 00 Lund, Sweden

## ○

NOAA Chemical Sciences Laboratory, 325 Broadway, R/CSL6, Boulder, Colorado 80305, United States, and Cooperative Institute for Research in Environmental Sciences, University of Colorado Boulder, Boulder, Colorado 80309, USA.
