# Supplementary material for: Core-Level Photoelectron Angular Distributions from Bulk-Solvated to Surface-Active Aqueous Potassium Carboxylate Salts
Source: J Phys Chem B. 2025 Sep 10;129(37):9430–8. doi: 10.1021/acs.jpcb.5c03673 (PMC12451660; doi:10.1021/acs.jpcb.5c03673)
Supplement: Supplementary file 1 [file jp5c03673_si_001.pdf]

Supporting Information: Core-Level Photoelectron Angular Distributions From Bulk-Solvated to Surface-Active Aqueous Potassium Carboxylate Salts

Tamires M. Gallo,[†] Kalil Cristhian Figueiredo Toledo,[‡] Georgia Michailoudi,[¶] Ricardo dos Reis Teixeira Marinho,[§,‖] Olle Björneholm,[⊥] Noelle Walsh,[#] and Gunnar Öhrwall[*,#]

†*Division of Synchrotron Radiation Research, Department of Physics, Lund University, Box 118, SE-221 00 Lund, Sweden*

‡*Institute of Chemistry, University of São Paulo - IQ-USP, Av. Lineu Prestes 748, 05508-000 São Paulo, SP, Brazil*

¶*Nano and Molecular Systems Research Unit, University of Oulu, P.O. Box 3000, FI-90014 Oulu, Finland*

§*Institute of Physics, Brasilia University (UnB), 70.910-900, Brasília, Brazil*

‖*Institute of Physics, Federal University of Bahia, 40.170-115, Salvador, BA, Brazil*

⊥*Department of Physics and Astronomy, Uppsala University, Box 516, SE-75120 Uppsala, Sweden*

#*MAX IV Laboratory, Lund University, Box 118, SE-22100 Lund, Sweden*

E-mail: gunnar.ohrwall@maxiv.lu.se

Details about the fitting of the spectra

To analyze the experimental C 1s and K 2p photoelectron spectra, we used the SPANCF fitting routines (described in Ref.[S1,S2]) for Igor Pro (WaveMetrics).[S3] The photoelectron peaks were described by symmetric Voigt profiles, where the Lorentzian lifetime widths were set to 0.1 eV for the C 1s peaks[S4] and 0.19 eV for the K 2p peaks,[S5] and the Gaussian widths were free parameters (constrained to be equal for the two spin-orbit components of K 2p). The errors for the peak areas were derived using the standard deviation of the fitting parameters estimated by the SPANCF fitting routine. The error bars in the figures are calculated from the propagation of the errors of the statistical uncertainties and are presented as 1.96 * (compound error). In all cases, the fits included a linear background and a weak and broad Gaussian peak to account for weak inelastic loss structures in the high-binding-energy region. For the lowest photon energy ($h\nu$=360 eV), an additional broad Gaussian peak was added in the high-binding-energy region to account for the nonlinearity in the background. At the highest photon energy ($h\nu$=550 eV), distinct features from K $L_{2,3}M_{2,3}M_{2,3}$ Auger decays are seen around the binding energy 305 eV, and two additional peaks were added to account for them. In the normalization procedure, we have used the linear background obtained from the fit, and with the additional peaks in the high-binding-energy region, this becomes robustly constrained by the low-binding-energy part of the spectrum, below the K $2p_{3/2}$ peak.

Estimate of orientation of molecules at the surface

The photoelectron signal is exponentially reduced by inelastic scattering as a function of distance from the surface, and by making an assumption about the mean free path of the electrons, the ratio of the carbon signals from the chain and the carboxylate carbon can inform about the orientation of the molecule. In gas phase, the straight-chained carboxylate ions have a distance between neighboring carbon atoms of ~1.52 Å and a dihedral angle of ~111°-115°, meaning the projected distance between neighboring carbons along the chain is

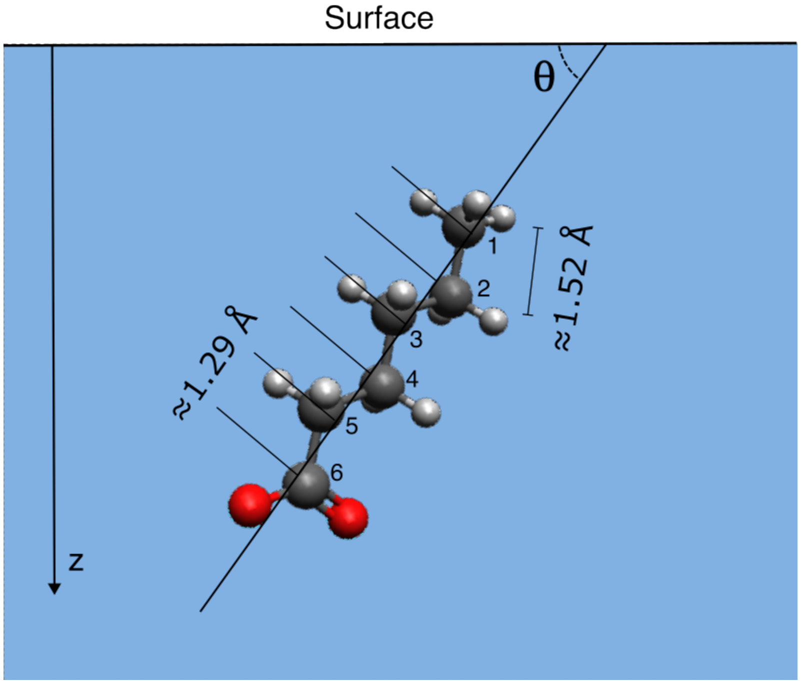

Figure S1: Sketch to illustrate the geometry used for the intensity ratio estimates. Note that the position of the molecule relative to the surface is unknown, but does not matter for the discussion below as long as the inelastic mean free path is the same for all atoms.

~1.52·sin(112°/2)=1.29 Å, as illustrated in Fig. S1 for the case of hexanoate.[S6] The C 1s photoelectron intensities for emission normal to the surface of the chain and the carboxylate can in a simple model be estimated as

$$\begin{aligned} \mathrm{I}_{\mathrm{C}_n\mathrm{H}_m} &= \sum_{i=1}^{5} \mathrm{I}_0 e^{-z_i/\lambda} \\ \mathrm{I}_{\mathrm{COO}^-} &= \mathrm{I}_0 e^{-z_6/\lambda}, \end{aligned} \tag{1}$$

where the carbon atoms have been numbered i =1-6, z_i is the distance to the surface of each carbon atom, λ is the inelastic mean free path (IMFP), and it is assumed that the photoionization cross section is the same for all carbon atoms. As an additional simplification, the distances to the surface (in Å) can be estimated as $z_i = z_1 + (i-1)\cdot 1.29 \cdot sin(\theta)$, where θ is the tilt angle of the molecule with respect to the surface. With λ given in units of Å,

the ratio of the intensities will then be

$$\frac{\mathrm{I}_{\mathrm{C}_n\mathrm{H}_m}}{\mathrm{I}_{\mathrm{COO}^-}} = \frac{\sum_{i=1}^{5} \mathrm{I}_0 e^{-z_i/\lambda}}{\mathrm{I}_0 e^{-z_6/\lambda}} = \sum_{i=1}^{5} e^{(z_6-z_i)/\lambda} \approx \sum_{i=1}^{5} e^{(6-i)\cdot 1.29\cdot sin(\theta)/\lambda}. \tag{2}$$

Note that for the ratio of the intensities, the absolute distance to the surface does not matter, only the difference in path for the atoms in the molecule (as longs as the IMFP is the same). Although the interatomic distances are well known, the IMFP of the electrons has some uncertainty, and the tilt angle with respect to the surface is unknown. It should be noted that if λ is longer than the molecule,

$$\sum_{i=1}^{5} e^{(z_6-z_i)/\lambda} \approx 5e^{(z_6-\overline{z_i})/\lambda}, \tag{3}$$

where $\overline{z_i}$ is the average of z_i (i=1-5). With the above assumptions about the positions of carbon atoms, $z_6 - \overline{z_i} = (6-3)\cdot 1.29 \cdot sin(\theta) \approx 3.9 \cdot sin(\theta)$ Å. The ratio $\mathrm{I}_{\mathrm{C}_n\mathrm{H}_m}/\mathrm{I}_{\mathrm{COO}^-} = 6.8 \approx 5e^{(z_6-\overline{z_i})/\lambda}$ gives $z_6 - \overline{z_i} = \lambda \cdot \ln(6.8/5) \approx 3.1$ Å for $\lambda = 10$ Å, and $\theta \approx \arcsin(3.1/3.9) \approx 52°$.

Experimental data tables

In the tables below, the measured areas of the C 1s peaks of the carboxylate group (COO^-) and aliphatic chains (C_nH_m) and of the potassium K $2p_{3/2}$ peak are presented, together with the calculated β values of the peaks. The areas have been normalized to the area of the K $2p_{3/2}$ peak for each energy recorded at 54.7°, which means that the data recorded at 90° have been normalized using the intensity of the background under the assumption that the background has an isotropic angular distribution, as described in the main text. The error in the β value has been calculated from the statistical errors obtained from the fitting procedure (see main text), and assuming an uncertainty of 1° (σ) in the angle and 0.01 (σ) in the degree of linear polarization. The errors are given as 1.96σ.

Table S1: K $2p_{3/2}$ and C 1s peak areas for potassium formate and calculated β values

hν (eV)	360	380	400	425	450	500	550
K $2p_{3/2}$ 54.7°	1	1	1	1	1	1	1
K $2p_{3/2}$ 90°	0.760	0.674	0.653	0.580	0.555	0.492	0.439
K $2p_{3/2}$ β	0.479	0.652	0.693	0.840	0.889	1.016	1.122
K $2p_{3/2}$ β error	0.157	0.142	0.139	0.125	0.121	0.111	0.104
$HCOO^-$ 54.7°	0.341	0.345	0.362	0.342	0.321	0.295	0.288
$HCOO^-$ 90°	0.141	0.134	0.131	0.104	0.099	0.086	0.080
$HCOO^-$ β	1.170	1.225	1.274	1.394	1.379	1.415	1.447
$HCOO^-$ β error	0.108	0.099	0.095	0.080	0.082	0.081	0.107

Table S2: K $2p_{3/2}$ and C 1s peak areas for potassium acetate and calculated β values.

hν (eV)	360	380	400	425	450	500	550
K $2p_{3/2}$ 54.7°	1	1	1	1	1	1	1
K $2p_{3/2}$ 90°	0.731	0.714	0.652	0.597	0.566	0.527	0.456
K $2p_{3/2}$ β	0.538	0.572	0.696	0.806	0.868	0.946	1.087
K $2p_{3/2}$ β error	0.151	0.148	0.137	0.129	0.123	0.116	0.105
COO^- 54.7°	0.365	0.382	0.384	0.341	0.324	0.297	0.290
COO^- 90°	0.143	0.134	0.129	0.113	0.098	0.084	0.070
COO^- β	1.219	1.299	1.329	1.331	1.392	1.432	1.521
COO^- β error	0.096	0.088	0.085	0.086	0.079	0.074	0.077
CH_3 54.7°	0.424	0.406	0.445	0.375	0.375	0.354	0.291
CH_3 90°	0.140	0.118	0.114	0.101	0.091	0.084	0.068
CH_3 β	1.342	1.421	1.487	1.462	1.516	1.527	1.529
CH_3 β error	0.082	0.077	0.069	0.074	0.066	0.064	0.092

Table S3: K $2p_{3/2}$ and C 1s peak areas for potassium propanoate and calculated β values.

hν (eV)	360	380	400	425	450	500	550
K $2p_{3/2}$ 54.7°	1	1	1	1	1	1	1
K $2p_{3/2}$ 90°	0.775	0.699	0.617	0.588	0.554	0.491	0.468
K $2p_{3/2}$ β	0.450	0.601	0.767	0.824	0.891	1.018	1.064
K $2p_{3/2}$ β error	0.158	0.145	0.132	0.127	0.121	0.110	0.113
COO^- 54.7°	0.379	0.385	0.398	0.357	0.340	0.335	0.349
COO^- 90°	0.160	0.155	0.131	0.110	0.099	0.087	0.093
COO^- β	1.159	1.193	1.340	1.384	1.421	1.480	1.466
COO^- β error	0.109	0.108	0.101	0.091	0.085	0.113	0.088
C_2H_5 54.7°	0.808	0.806	0.852	0.896	0.877	0.900	0.940
C_2H_5 90°	0.288	0.241	0.217	0.193	0.171	0.148	0.145
C_2H_5 β	1.377	1.471	1.507	1.533	1.580	1.607	1.614
C_2H_5 β error	0.077	0.067	0.065	0.062	0.057	0.053	0.073

Table S4: K $2p_{3/2}$ and C 1s peak areas for potassium butanoate and calculated β values.

hν (eV)	360	380	400	425	450	500	550
K $2p_{3/2}$ 54.7°	1	1	1	1	1	1	1
K $2p_{3/2}$ 90°	0.820	0.692	0.643	0.609	0.547	0.497	0.487
K $2p_{3/2}$ β	0.360	0.616	0.713	0.781	0.907	1.006	1.026
K $2p_{3/2}$ β error	0.168	0.146	0.138	0.132	0.120	0.113	0.124
COO^- 54.7°	0.457	0.461	0.446	0.410	0.390	0.366	0.409
COO^- 90°	0.216	0.154	0.153	0.124	0.106	0.088	0.114
COO^- β	1.051	1.334	1.315	1.394	1.456	1.517	1.441
COO^- β error	0.129	0.101	0.105	0.100	0.085	0.086	0.182
C_3H_7 54.7°	1.760	1.649	1.606	1.518	1.456	1.328	1.357
C_3H_7 90°	0.562	0.398	0.374	0.321	0.290	0.236	0.216
C_3H_7 β	1.362	1.517	1.534	1.576	1.602	1.645	1.682
C_3H_7 β error	0.078	0.062	0.060	0.056	0.052	0.048	0.054

Table S5: K $2p_{3/2}$ and C 1s peak areas for potassium hexanoate and calculated β values.

hν (eV)	360	380	400	425	450	500	550
K $2p_{3/2}$ 54.7°	1	1	1	1	1	1	1
K $2p_{3/2}$ 90°	0.767	0.618	0.569	0.542	0.549	0.478	0.442
K $2p_{3/2}$ β	0.466	0.764	0.861	0.915	0.902	1.043	1.115
K $2p_{3/2}$ β error	0.161	0.140	0.128	0.123	0.127	0.112	0.142
COO^- 54.7°	0.615	0.595	0.548	0.518	0.498	0.491	0.661
COO^- 90°	0.228	0.164	0.159	0.141	0.122	0.125	0.147
COO^- β	1.259	1.448	1.421	1.454	1.509	1.492	1.555
COO^- β error	0.118	0.109	0.106	0.100	0.095	0.158	0.684
C_5H_{11} 54.7°	4.195	3.956	3.581	3.453	3.407	3.132	2.969
C_5H_{11} 90°	1.191	0.813	0.656	0.628	0.559	0.453	0.384
C_5H_{11} β	1.432	1.589	1.634	1.636	1.672	1.711	1.741
C_5H_{11} β error	0.069	0.053	0.047	0.047	0.043	0.039	0.054

References

(S1) Kukk, E.; Snell, G.; Bozek, J. D.; Cheng, W.-T.; Berrah, N. Vibrational Structure and Partial Rates of Resonant Auger Decay of the N $1s \rightarrow 2\pi$ Core Excitations in Nitric Oxide. *Phys. Rev. A* **2001**, *63*, 062702.

(S2) Kukk, E.; Ueda, K.; Hergenhahn, U.; Liu, X.-J.; Prümper, G.; Yoshida, H.; Tamenori, Y.; Makochekanwa, C.; Tanaka, T.; Kitajima, M. et al. Violation of the Franck-Condon Principle due to Recoil Effects in High Energy Molecular Core-Level Photoionization. *Phys. Rev. Lett.* **2005**, *95*, 133001.

(S3) *Igor Pro*, version 8.04; WaveMetrics, Inc.: Lake Oswego, OR, 2019.

(S4) Campbell, J. L.; Papp, T. Widths of the Atomic K-N7 Levels. *At. Data Nucl. Data Tables* **2001**, *77*, 1–56.

(S5) Kukk, E.; Huttula, M.; Aksela, H.; Akela, S.; Nõmmiste, E.; Kikas, A. Limitations of the Ionic Model in Describing Core-Hole Decay - Molecular Versus Crystalline KCl. *J. Phys. B: At. Mol. Opt. Phys.* **2003**, *36*, L85.

(S6) National Center for Biotechnology Information. PubChem Compound Summary for CID 4398339, Hexanoate. https://pubchem.ncbi.nlm.nih.gov/compound/Hexanoate. Accessed Mar. 26, 2025.
